# Demographic, exposure, clinical, biochemical and diagnostic data of febrile patients recruited for the largest field study on leptospirosis in Sri Lanka

**DOI:** 10.1016/j.dib.2022.108378

**Published:** 2022-06-12

**Authors:** Suneth Agampodi, Janith Warnasekara, Sisira Siribaddana, SAM Kularatna, Chandika Gamage, Dinesha Jayasundara, Indika Senevirathna, Shalka Srimantha, Chamila Kappagoda, Prasanna Weerawansa, Senaka Pilapitiya, Niroshan Lokunarangoda, Chamara Sarathchandra, Hemal Senanayake, Michael Matthias, Joseph Vinetz

**Affiliations:** a Department of Community Medicine, Faculty of Medicine and Allied Sciences, Rajarata University of Sri Lanka, Sri Lanka; b Department of Medicine, Faculty of Medicine and Allied Sciences, Rajarata University of Sri Lanka, Sri Lanka; c Department of Medicine, Faculty of Medicine, University of Peradeniya, Sri Lanka; d Department of Microbiology, Faculty of Medicine, University of Peradeniya, Sri Lanka; e Department of Microbiology, Faculty of Medicine and Allied Sciences, Rajarata University of Sri Lanka, Sri Lanka; f Department of Biochemistry, Faculty of Medicine and Allied Sciences, Rajarata University of Sri Lanka, Sri Lanka; g Section of Infectious Diseases, Department of Internal Medicine, School of Medicine, Yale University, United States

**Keywords:** *Leptospira*, Leptospirosis, Microscopic agglutination test, qPCR

## Abstract

This dataset includes data from febrile patients recruited for a large hospital-based study in Sri Lanka from 2016 to 2019. The variables include primary socio-demographic data, exposure data, clinical data, biochemical and investigation data. Some of these data are available as serial data from admission to discharge daily. Microscopic agglutination test, quantitative PCR of whole blood, urine and serum and culture isolation was performed to diagnose the patients with leptospirosis.

## Specifications Table

**Table:** 

Subject	Infectious Disease
Specific subject area	Clinical, epidemiological and diagnostic data on leptospirosis.
Type of data	Table
How the data were acquired	Data were acquired through A hospital-based surveillance system: all demographic, clinical and some of the biochemical data were acquired through an interviewer administrated questionnaire and a data extraction format. Some of the blood biochemistry data were derived from the analysis of the collected sample using a fully automated biochemistry analyser. qPCR data were obtained through a quantitative PCR equipment MAT data were generated through a selected MAT panel
Data format	Raw and filtered
Description of data collection	Data were collected by trained clinical interviewers (either nursing or medical graduates). Febrile patients admitted to selected hospitals were screened, enrolled and followed up.
Data source location	Institution: Rajarata University of Sri Lanka City/Town/Region: Saliyapura, North Central province Country: Sri Lanka
Data accessibility	The dataset is available from https://data.mendeley.com/datasets/zmxmp42g9p/4 Name of the dataset: Demographic, exposure, clinical, biochemical and diagnostic data of febrile patients recruited for the largest field study on leptospirosis in Sri Lanka DOI: 10.17632/zmxmp42g9p.4 Files available: The dataset in spss format .sav Dataset in CSV format .csv Codebook for CSV data Tools used I. Symptoms and signs on admission Tools used II. Event calendar Tools used II. Questionnaire

## Value of the Data

• This dataset provides sequential data on clinical and biochemical parameters of leptospirosis patients with their diagnosis and microscopic agglutination test results using a broad panel of serovar. Leptospirosis investigation lacks this type of comprehensive clinical data to understand the clinical spectrum of disease. In addition, exposure data are also included for a subsample.
• Those who have been involved in leptospirosis research and clinical practice and all other researchers in infectious disease, epidemiology, and public health could benefit from these data since our data are comparable with all other infectious disease data.
• This dataset can be used to compare and contrast the different clinical phenotypes of leptospirosis, plan clinical studies, and use hypothesis generation and testing.
• Since the sequential clinical data are provided in this dataset, clinical studies from different geographical locations could be planned based on this work.

## 1 Data Description

The dataset included in this article is presented as a single table with all deidentified patient data. Each row contains a single patient. Depending on the date of hospital admission, date of discharge, method of recruitment and the investigations requested by the physicians, several missing values are present.

## 2 Experimental Design, Materials and Methods

The initial study protocol for this data was published in 2019. The MAT data, the experimental design, material and methods were also published in the protocol paper and subsequent results papers [1], [2], [3], [4].

### 2.1 Study setting

This study was carried out in selected hospitals in Sri Lanka. The data presented in this paper were acquired through different mechanisms. Teaching Hospital Anuradhapura (THA) and Teaching Hospital Peradeniya (THP) were the primary research sites for the study, in which the data collection started in 2016. During the 2017 flood season, Base Hospital Awissawella (BHA) and Provincial General Hospital Rathnapura (PGHR) were also included in the study. The District General Hospital in Polonnaruwa provided us with routine samples from possible leptospirosis patients for diagnosis (DGHP). We acquired culture samples from typical instances of clinical leptospirosis from District General Hospital Kegalle (DGHK), Base Hospital Karawanella (BHK), and Sri Jayawardanapura General Hospital (SJGH) for retrospective disease confirmation.

### 2.2 Study participants, sample and data collection

Clinically trained data collectors visited all internal medical wards, intensive care units and special units where probable cases of leptospirosis patients could be admitted, and all undifferentiated febrile patients (temperature >38 °C and fever less than 15 days) were interviewed. Once the inclusion and exclusion criteria were met, those patients were enrolled in the study after written informed consent. Patients with physician-diagnosed probable or definite acute bacterial meningitis or lower respiratory tract infections (e.g., consolidated lobar pneumonia), traumatic or post-operative fever, fever due to nosocomial infections, and any patient with the confirmed diagnosis of a cause for the fever were all excluded. At the time of recruitment, a baseline assessment was done using a symptom checklist that included all clinical symptoms from the date of onset of the disease until the interview date. A clinical examination was also performed. Exposure data were collected through a previously validated questionnaire for leptospirosis investigation. Routinely available investigation results with patient records were also extracted. These patients were visited daily in the ward until discharge and the clinical features and investigation results were updated daily. Patients were requested to visit the hospital 14 days after the discharge and the clinical symptom checklist was updated during this period. A serum and urine samples were obtained during the follow-up visit as well.

Biological samples were taken for serological, molecular and blood biochemistry testing and for culture isolation. The sample includes a whole blood sample, serum sample, and urine sample. All collected samples were processed on-site and transported to the Public Health Research Laboratory of Faculty of Medicine and Allied Sciences, Rajarata University of Sri Lanka. All testing, culture and storage were done as per the previously published protocols.

### 2.3 Laboratory procedures

For MAT, fresh blood was drawn to a plain tube from the eligible individuals and allowed a clot to be formed at room temperature before serum separation. 500 ul aliquots were made and stored at −20 °C and −80 °C, respectively, for short and long-term storage. This study used the MAT panel developed by the US Centers for Disease Control and Prevention, which included WHO-recommended serovars(1). In addition, strains isolated from Sri Lanka were obtained from the Department of Medical Microbiology of the Academic Medical Centre (AMC) in Amsterdam and included (Table 1). The MAT procedure was done using the standard protocol described in the published article [2]. Only a single antiserum was used as a positive control.

**Table 1:** Basic characteristics of the data set.

Variable	Sub category	Value
Number of Patients	Total Undifferentiated febrile patients Clinically suspected (Requested by physicians) Paediatric Patients (Requested by physicians)	1734 (100%) 1513 (87.3%) 207 (11.9%) 14 (0.8%)
Age	Minimum Maximum Mean SD Missing	2 years 87 years 43.3 years 14.7 years 213 (12.2%)
Gender	Male Female Missing	1258 (72.5%) 298 (17.2%) 178 (10.3%)
Section of the Hospital	Out-Patient Inward Missing	92 (5.3%) 1543 (89%) 99 (5.7%)
Data availability	Epidemiological data Clinical data Biochemical data	1348 (77.7%) 1293 (74.6%) 1180 (68.1%)
Sample Availability	Whole Blood Serum Urine Follow up Whole blood Culture	1455 (83.9%) 352 (20.3%) 498 (28.4%) 302 (17.4%) 1192 (20.3%)

DNA extraction for qPCR was done from 200 µL of whole blood using QIAamp DNeasy Mini kit (Qiagen, Germany). Amplification of the target gene was carried out in 20 µL of total reaction volume containing 10 µL of PerfeCTa SYBR Green FastMix (Quanta bio, USA), 0.02 µL of each primer (100 µM), 5 µL of template DNA (concentration varies from 8 to 10 ng/µL), and 4.96 µL of nuclease-free water. qPCR was performed using the real-time PCR detection system CFX96 (Bio–Rad, USA). The set of primer pairs targeting 16s rRNA gene included; Forward-5′-GCGTAGGCGGACATGTAAGT-3′ and Reverse-5′-AATCCCGTTCACTACCCACG-3′. For the LipL32 gene; Forward-5′ TGG CTA TCT CCG TTG CAC TC 3′, Reverse-5′ CCC ATT TCA GCG ATT ACG GC 3′ [5]. Thermal cycle conditions used with 16S primer pair were as: 95 °C for 5 min, 45 cycles of [94 °C for the 30s,60 °C for 30 s], followed by a melt curve from 65 °C to 90 °C performed at an increment of 0.5 °C per cycle. Thermal cycle conditions used with LipL32 primer pair were: 95 °C for 2 min, 45 cycles of[95 °C for 5 s,60 °C for 35 s], followed by a melt curve from 65 °C to 90 °C performed at an increment of 0.5 °C per cycle [5].

Blood for culture was performed by collecting 2–4 drops of blood into EMJH semisolid media.

Blood biochemistry testing of collected samples was carried out using Mindray BS-240 Clinical Chemistry Analyzer (Table 2).

**Table 2:** *Leptospira* strains included in the MAT panel.

*L. interrogans* serovar Bratislava str. Jez-Bratislava
*L. interrogans* serovar Canicola str. Ruebush
*L. interrogans* serovar Weerasinghe str.Weerasinghe
*L. interrogans* serovar Icterohaemorrhagiae str. RGA
*L. santarosai* serovar Georgia str. LT 117
*L. interrogans* serovar Bataviae str. Van Tienan
*L. interrogans* serovar Mankarso str. Mankarso
*L. interrogans* serovar wolfii str. 3705
*L. borgpetersenii* serovar Ceylonica str. Piyasena
*L. interrogans* serovar Pomona str. Pomona
*L. santarosai* serovar Pyrogenes str. Salinem
*L. weilii* serovar Celledoni str. Celledoni
*L. interrogans* serovar Alexi str. 616
*L. interrogans* serovar Autumnalis str. Akiyami A
*L. borgpetersenii* serovar Ballum str. Mus 127
*L. interrogans* serovar Djasiman str. Djasiman
*L. interrogans* serovar Australis str. Ballico
*L. kirschneri* serovar Ratnapura str. Wumalasena
*L. borgpetersenii* serovar tarassovi str. Perepelitsyn
*L. santarosai* serovar Alice str. Alice
*L. interrogans* serovar Grippotyphosa
*L. interrogans* serovar Geyaweera str. Geyaweera
*L. santarosai* serovar Borincana str. HS 622
*L. biflexa* serovar Patoc str Patoc 1

## Ethics Statements

Written informed consent was obtained from all patients or guardians (for minors) included in this study. Ethical clearance was obtained from the Ethics Review Committee of Faculty of Medicine and Allied Sciences, Rajarata University of Sri Lanka. (ERC/2015/18).

## CRediT Author Statement

**Suneth Agampodi:** Conceptualization, Funding acquisition, Methodology, Design, Project administration, Data curation, Supervision, Writing – original draft preparation; **Janith Warnasekara:** Designing field data collection, Data collection, Data curation, Investigation; **Dinesha Jayasundara:** Investigation (culture isolation and MAT), data curation; **Indika Senevirathna:** Investigation, data curation; **Sisira Siribaddana and SAM Kularatne:** Supervision of clinical data collection, Clinical investigations; **Chandika Gamage:** Investigations, supervision; **Prasanna Weerawansa, Senaka Pilapitiya, Niroshan Lokunarangoda, Chamara Sarathchandra** and **Hemal Senanayaka:** Clinical Investigations; **Shalka Srimantha and Chamila Kappagoda:** Investigations (qPCR), software and data curation; **Michael Matthias:** Data validation, design; **Joseph Vinetz:** Conceptualization, Funding acquisition, Supervision, Writing – editing the original draft.

## Declaration of Competing Interest

The authors declare that they have no known competing financial interests or personal relationships that could have appeared to influence the work reported in this paper.
